# Comparing Ensemble and Standalone Machine Learning Models for Optimizing Biodiesel Production via Esterification

**DOI:** 10.1021/acsomega.6c04115

**Published:** 2026-06-26

**Authors:** Melike İmge Şenoymak Tarakçı

**Affiliations:** Faculty of Engineering (B), Chemical Engineering Department, Kocaeli University, Umuttepe Campus, 41380 İzmit, Kocaeli, Turkey

## Abstract

The increasing energy demand and environmental problems caused by fossil fuel consumption have increased the demand for sustainable and green energy sources. Biofuels stand out as alternative fuels that are renewable, biodegradable, and environmentally friendly. However, traditional experimental methods used in biofuel production are restrictive in both time and cost in determining and optimizing process parameters. Therefore, modeling approaches based on machine learning (ML) techniques have recently stood out to improve production efficiency. In this study, the biodiesel production process based on esterification reaction of oleic acid was modeled and optimized by using four distinct models—**Multilayer Perceptron (MLP)**, **AdaBoost SVR**, **Gradient Boosting**, and a high-performance **Voting Regressor** (an ensemble of the three). The model utilizes key process parameters including reaction time (1–6 h), catalyst loading (3–18% by weight), and a methanol/oleic acid molar ratio (3:1–15:1) to predict oleic acid conversion (%). Model performances were evaluated using *R* ^2^, mean absolute percentage error (MAPE), and mean square error (MSE) values obtained from training, testing, and cross-validation data sets. Among the models, the Voting Regressor achieved the highest test *R* ^2^ value (0.9653), with relatively low test MAPE (2.27%) and MSE (4.7557) values, as well as the highest cross-validation *R* ^2^ value (0.9513). Parametric analysis using the Voting Regressor model showed that increasing catalyst loading and methanol/oleic acid molar ratio improved conversion up to a certain level, after which a plateau tendency was observed. Under optimized conditions (5.49 h, 18 wt % catalyst, and 15:1 molar ratio), a maximum conversion of 99.23% was predicted. The results demonstrate that ensemble learning can significantly improve the reliability of biodiesel process modeling while reducing experimental workload in chemical engineering applications.

## Graphical abstract

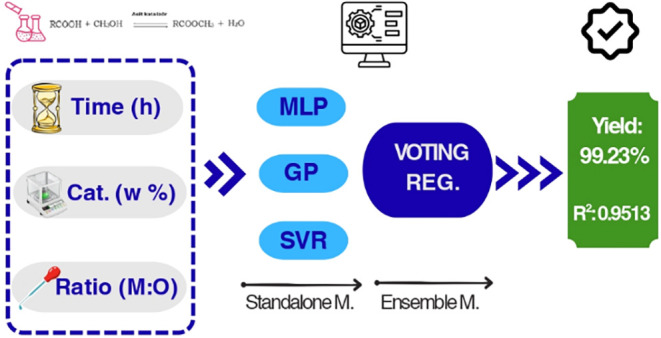

## 1 Introduction

The energy crisis following the heavy dependence on high-cost fossil fuels due to their nonrenewable and scarce nature has accelerated the search for alternative fuels that are renewable and sustainable energy sources.^1,2^ Biodiesel attracts worldwide attention as an environmentally friendly and biobased fuel compared to petroleum diesel. As presented in Figure 1, global biofuel production has shown a remarkable increase across different regions as a result of the growing demand for renewable and environmentally friendly energy sources. This continuous increase in global biofuel production also reflects the growing importance and demand for biodiesel and other renewable fuels.

**Figure 1:**
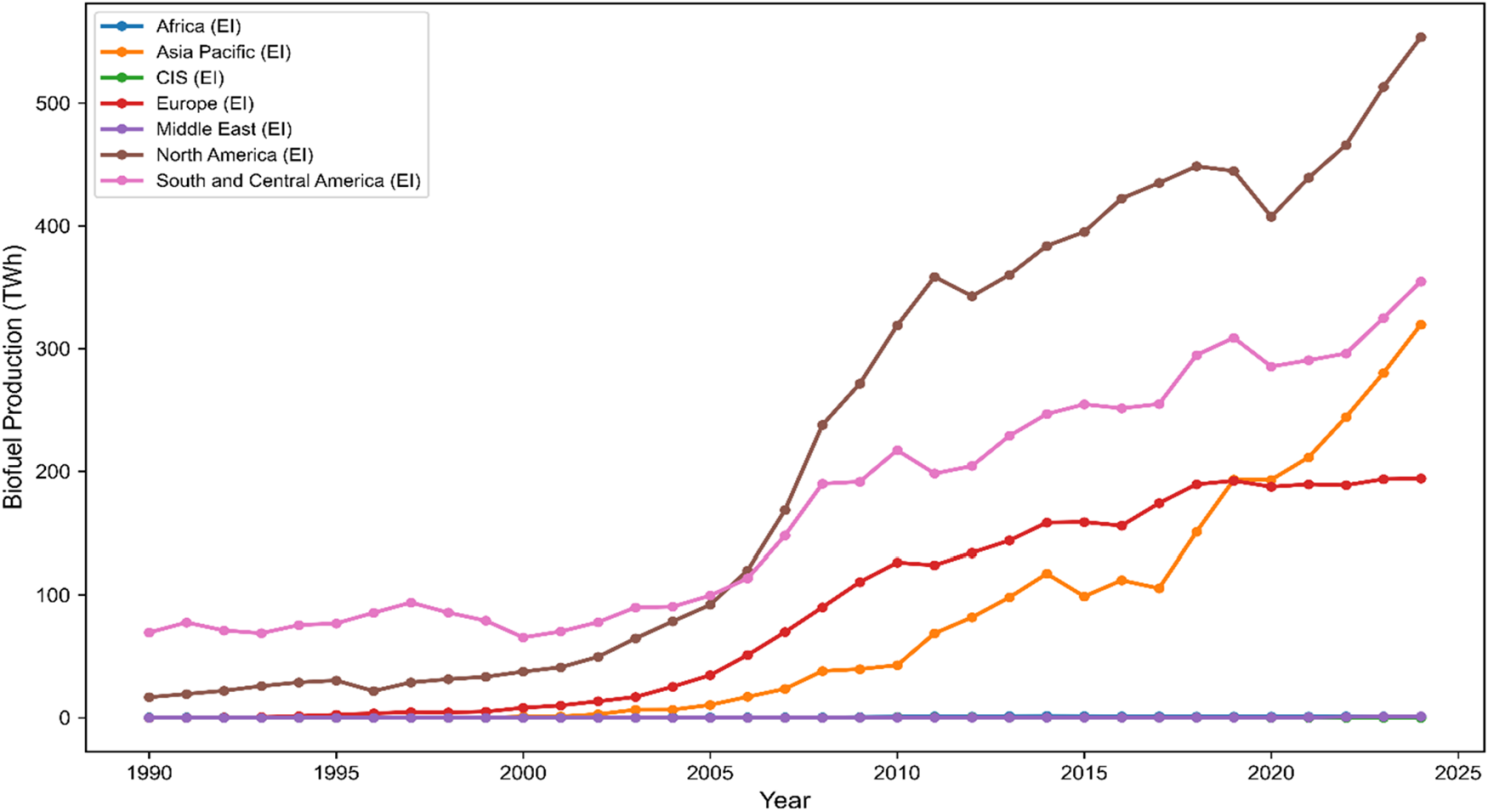
Global biofuel production trends by region over recent decades adapted from ref 3. Source: Energy Institute—Statistical Review of World Energy (2025)—with major processing by Our World in Data (CC BY).

Biodiesel, also called fatty acid alkyl ester, is basically obtained by transesterification of triglycerides from vegetable and animal oils or by esterification of fatty acids with alcohols such as methanol or ethanol in the existence of an alkali or acid catalyst.^4^ However, high production cost poses an obstacle to the large-scale commercialization of biodiesel.^5^ As a solution to this problem, vegetable oils and animal fats have gained importance as cheaper, promising, and easily accessible raw materials in biodiesel production. While alkaline catalysts (KOH, NaOH) are generally used for oils containing low free fatty acids (FFA), in situations where the feedstock has high FFA content, acid catalysts are required.^6,7^ In addition, acid-catalyzed esterification can also be applied to reduce the FFA content. On the other hand, homogeneous acid and alkali catalysts have some disadvantages, such as difficulties in catalyst separation from reaction medium, side reactions, nonreusability, and corrosion.^8–10^ In order to overcome the mentioned problems, ion-exchange resins,^11,12^ carbon-based catalysts,^13,14^ heteropoly acids,^15,16^ zeolites,^17,18^ and catalyst supports^19,20^ are recommended.

Among these, ion-exchange resins have become prominent because they can catalyze the reaction under milder conditions due to their high acid concentrations, high selectivity, environmental friendliness, and easy commercial availability.^21,22^ In recent years, many studies have been presented about the use of these resins in biodiesel production.^23–26^ However, research on the Purolite CT169DR catalyst, one of the ion-exchange resins, remains limited, especially in the context of esterification and transesterification reactions.^27–29^

The main parameters affecting the biodiesel yield are the type of catalyst used, the oil-to-alcohol molar ratio, the amount of catalyst used, reaction time, and temperature.^30^ The experimental investigation of the importance of these factors and their effects on the biodiesel production process is reported in a wide range of published studies.^31–34^ Although extensive experimental studies have been performed to analyze the effect of these factors, traditional approaches frequently require time-consuming and excessive cost experimental processes.^35^ Additionally, the complex interactions among these variables make it difficult to reach general conclusions. In this regard, machine learning (ML) methods appeared as powerful tools in modeling and optimization for biodiesel production. As a result of using machine learning (ML) algorithms, researchers can predict optimizable reaction conditions and key parameters more accurately and efficiently that affect the biodiesel production process compared to traditional methods.^36^

In recent years, ML, as a new type of analysis technique, has gained attention in the computational chemistry field such as molecular and materials engineering,^37^ chemical engineering,^38^ bioprocess,^39^ and energy applications.^2,40^ Moreover, new application areas that use ML are expanding day by day.^41,42^ Consisting of reliable and advanced data analysis methods, ML technology is a versatile approach that relates inputs to their corresponding outputs based on mathematical and probability/statistics principles. ML enables making predictions when new input is provided by recognizing relationships between features in a data set.^2^

ML has the potential to bring extensive innovations to the industry when used in the biodiesel production process. ML algorithms give an outstanding performance in determining the best options by analyzing various factors such as cost, availability, and oil content about raw material selection and parametric optimization. ML supports the quality control processes by analyzing biodiesel samples and plays an active role in meeting the product’s industry standards. Also, ML-based troubleshooting and maintenance processes prevent production breakdowns by early equipment failures detection, increase efficiency, and reduce maintenance costs. Furthermore, predictive modeling helps analyze different feedstock combinations and process parameters to determine biodiesel yield and quality. ML optimizes the transesterification and esterification reactions processes by adjusting parameters such as temperature, pressure, catalyst loading, alcohol-to-oil molar ratio, and reaction time while maximizing yield and minimizing energy consumption and byproducts. ML-based models can predict biodiesel yield by learning from experimental data and show high prediction performance to determine the optimum production parameters. However, the diversity of feedstocks used in biodiesel production and the reaction parameters variations in different systems may limit the generalizability of ML models. Therefore, it is important to predict and comparatively evaluate biodiesel conversion efficiency using different ML methods. In general, ML integration supports improvements in the renewable energy sector while enhancing quality and sustainability in biodiesel production.^2,43–45^

Recent studies have demonstrated the increasing application of machine learning (ML) techniques in biodiesel production modeling and process optimization. Various ML algorithms, including ANN, MLP, SVR, Random Forest, XGBoost, Gradient Boosting, AdaBoost-based models, Gaussian Process Regression (GPR), and ensemble learning approaches, have been successfully applied to predict biodiesel yield and optimize reaction conditions in the transesterification process. Sivakumar et al. and Sukpancharoen et al. demonstrated that boosting-based models, particularly XGBoost, provided higher predictive accuracy than conventional ML methods for biodiesel yield prediction.^43,46^ Similarly, Liu and Zhang and also Tang et al. showed that ensemble tree-based methods such as Gradient Boosting and AdaBoost could accurately make prediction and optimization of biodiesel production by achieving *R* values above 0.99.^47,48^ In addition, Jin et al., Sumayli, and Sun et al. applied different ML techniques including ANN, MLP, KNN, GPR, and Gradient Boosting in biodiesel production studies from various raw materials and found high predictive accuracy with successful optimization of reaction parameters.^49–51^ Also, in another study of Jin et al., KNN, SVM, Random Forest, and AdaBoost regression models were applied to biodiesel production data sets obtained from different transesterification systems. Among these models, the random forest model achieved the highest predictive capability and demonstrated strong potential of ensemble-based ML approaches for biodiesel process optimization.^2^ In a separate study, Santos et al. revealed that combining several ML models with genetic algorithm optimization provides an efficient way for minimizing experimental time and operational costs in biodiesel production systems.^52^

Although a number of machine learning studies have investigated biodiesel production through transesterification processes by using different catalysts, studies focused on oleic acid esterification together with comparative ensemble machine learning modeling approaches remain limited in the literature. Furthermore, studies involving regression-based parametric analysis and comparative evaluations by cross-validation data are still limited in the literature. Accordingly, the primary objective of this study was to determine a reliable machine learning model for the prediction and optimization of FFA conversion during oleic acid esterification.

Herein, FFA conversion in the esterification reaction was analyzed using Optuna-optimized AdaBoost SVR, Bagging AdaBoost SVR, Gradient Boosting, Optuna-optimized double-layer Multilayer Perceptron (MLP), and Voting Regressor methods. The effects of main parameters, such as reaction temperature, catalyst amount, reaction time, and the methanol-to-oleic acid molar ratio, on FFA conversion were investigated, and the most suitable ML model was determined. The predictive performances of the models were compared using evaluation criteria such as *R* ^2^, mean absolute percentage error (MAPE), and mean square error (MSE). Thus, this study aims to improve the esterification process efficiency and reduce experimental time with cost by combining ensemble learning with regression-based parametric analysis. Thereby, it provides a novel, reliable, and data-driven framework for esterification-based biodiesel process optimization and decision-making.

## 2 Materials and Methods

### 2.1 Data Set

The experimental data of the esterification process used in biodiesel production in this study were obtained from our experimental study conducted previously.^29^ The data set includes key parameters affecting oleic acid conversion in the esterification reaction. This data set consisting of four numerical input and one numerical output variable is used. These are reaction temperature 67 °C, catalyst amount (wt %) ranging from 3 to 18, reaction time (h) ranging from 1 to 5, and methanol-to-oleic acid molar ratio ranging from 3/1 to 15/1. All data shown in Table 1 were obtained from 61 experiments and were used for the prediction and optimization of oleic acid conversion.

**Table 1:** Experimental Data Set

RUN	*X*_1_ = temperature (°C)	*X*_2_ = reaction time (h)	*X*_3_ = catalyst loading (wt %)	*X*_4_ = methanol/oleic acid molar ratio	*Y* = oleic acid conversion (%)
1	67	1	3	6/1	54.45
2	67	2	3	6/1	62.9
3	67	3	3	6/1	65.96
4	67	4	3	6/1	69.52
5	67	5	3	6/1	71.94
6	67	1	4.5	6/1	58.74
7	67	2	4.5	6/1	65.31
8	67	3	4.5	6/1	71.03
9	67	4	4.5	6/1	75
10	67	5	4.5	6/1	76.69
11	67	1	6	6/1	63.1
12	67	2	6	6/1	66.45
13	67	3	6	6/1	74.49
14	67	4	6	6/1	77.05
15	67	5	6	6/1	82.78
16	67	1	7.5	6/1	64
17	67	2	7.5	6/1	72.41
18	67	3	7.5	6/1	76.5
19	67	4	7.5	6/1	79.52
20	67	5	7.5	6/1	82.01
21	67	1	9	6/1	67.94
22	67	2	9	6/1	75.7
23	67	3	9	6/1	80.35
24	67	4	9	6/1	85.04
25	67	5	9	6/1	90.62
26	67	6	9	6/1	84.13
27	67	1	12	6/1	70.95
28	67	2	12	6/1	78.98
29	67	3	12	6/1	84.05
30	67	4	12	6/1	87.67
31	67	5	12	6/1	93.07
32	67	1	15	6/1	73.41
33	67	2	15	6/1	83.03
34	67	3	15	6/1	86.3
35	67	4	15	6/1	90
36	67	5	15	6/1	93.5
37	67	1	18	6/1	75.15
38	67	2	18	6/1	83.05
39	67	3	18	6/1	87.88
40	67	4	18	6/1	90.37
41	67	5	18	6/1	91.35
42	67	1	9	3/1	57.45
43	67	2	9	3/1	65.47
44	67	3	9	3/1	70.38
45	67	4	9	3/1	71.8
46	67	5	9	3/1	72.74
47	67	1	9	9/1	72.42
48	67	2	9	9/1	79.16
49	67	3	9	9/1	83
50	67	4	9	9/1	85.45
51	67	5	9	9/1	90.42
52	67	1	9	12/1	75.61
53	67	2	9	12/1	81.73
54	67	3	9	12/1	85.31
55	67	4	9	12/1	88.17
56	67	5	9	12/1	90.83
57	67	1	9	15/1	79.23
58	67	2	9	15/1	84.48
59	67	3	9	15/1	87.73
60	67	4	9	15/1	90.24
61	67	5	9	15/1	92.32

### 2.2 Modeling of Process

In this study, Optuna-optimized AdaBoost SVR, Bagging AdaBoost SVR, Gradient Boosting, Optuna-optimized dual-layer MLP, and Voting Regressor models were used. Machine learning algorithms were executed using the scikit-learn library in the Python 3.13.0 environment. The experimental data set used in the study was randomly divided into 80% training and 20% test subgroups.

#### 2.2.1 Adaptive Boosting Support Vector Regression (AdaBoost SVR)

AdaBoost (Adaptive Boosting) is a sequential ensemble learning method that works by creating multiple weak learners using various randomly selected subsets from the training data set. Initially, the algorithm assigns equal weights to all examples and then increases the accuracy of each weak learner by giving more importance to incorrectly predicted examples. In this way, it improves the overall model performance.^53,54^

The data points closest to the hyperplane are called support vectors and play a significant role in determining the location of the hyperplane. Support vector regression (SVR) is a version of support vector machines (SVM) adapted for regression and has the ability to provide robust predictions when working with high-dimensional data sets. The basic principle of SVR is to generate a hyperplane that provides the best fit while keeping the prediction error within a certain tolerance. The advantage of SVR is presenting high generalization ability even working with small data set and providing flexibility by using kernel functions for nonlinear data sets.^46,55^

The AdaBoost algorithm frequently uses Decision Trees (DTs) as weak learners; however, in this study, SVR is used as the base model. This method has been researched in studies in the literature and is applied for the first time in the biodiesel production process (transesterification and esterification) in this study. The AdaBoost SVR model begins by first assigning equal weights to all training data. Then, the initial SVR model is applied to the data set and predictions are made. To evaluate the accuracy of these predictions, the prediction error is calculated for each data point. The incorrectly predicted data points are updated to get higher weights. By this means, the model focuses more on previously incorrectly predicted examples in the next iteration. A new SVR model is trained with the updated weight, and this process continues until reaching the specified number of models. Lastly, the predictions of all individual SVR models are combined by taking a weighted sum and final prediction model is generated. The performance of the AdaBoost SVR model was measured using various regression performance metrics. These metrics are the following: “the Coefficient of Determination (*R* ^2^) indicates the accuracy with which the model explains the data,” “Mean Absolute Percentage Error (MAPE) measures the percentage error rate between the actual values and predicted values,” and “Root Mean Square Error (RMSE) represents the overall magnitude of error in the model’s predictions.” These performance metrics are used to assess the accuracy of the model and determine the best-performing parameters.

In this study, two different methods were tested separately to improve the performance of the AdaBoost SVR model. First, the Optuna method was used to perform hyperparameter optimization of the model and determine the best configuration. Then, the Gagging method was used to increase the stability of the model and reduce variance errors. Finally, the two approaches were compared.

The AdaBoost regression improves the prediction model by using a weighted prediction model.

When SVR is used as a regressor, the model can be formulated as follows:

First, the weighted error is calculated as follows

1 $$E_{m}=\Sigma_{i=1}^{N}w_{i}^{m}L(y_{i},f_{m}\left(x_{i}\right))$$

where *w*_*i*_^*m*^ is the weight of the *i*-th sample in the *m*-th iteration, *L*(*y*_*i*_, *f*_*m*_(*x*_*i*_)) represent loss function.

Then, the weight of the weak learner is calculated as

2 $$\alpha_{m}=\frac{1}{2}\ln\frac{1-E_{m}}{E_{m}}$$

where *E_m_* is the error ratio.

Sample weights are updated as follows

3 $$w_{i}^{m+1}=w_{i}^{m}e^{-\alpha_{m}y_{i}f_{m}\left(x_{i}\right)}$$

Final prediction model is obtained as the total of weighted SVR models

4 $$F_{x}=\Sigma_{m=1}^{M}\alpha_{m}f_{m}\left(x\right)$$

In the AdaBoost SVR model optimized with Optuna, hyperparameters such as n_estimators, learning_rate, C, epsilon, and loss function are optimized.

The following formula gives hyperparameter optimization

5 $$\Theta^{*}=\underset{\;}{\underset{\Theta }{\arg\min}\Sigma_{i=1}^{N}}L(y_{i},F_{\Theta }\left(x_{i}\right))$$

where Θ are the best values of hyperparameters such as n_estimators, learning_rate, C, epsilon, and loss that optimize by Optuna and *L*(*y*_*i*_,*F*_Θ_(*x*_*i*_)) is the selected error function (for example, Mean Square Error (MSE)).

Optuna determines these parameters via a Bayesian optimization.

The optimized regressor is adapted to the AdaBoost model as follows

6 $$F_{\mathrm{optuna}}\left(x\right)=\Sigma_{m=1}^{M}\alpha_{m}f_{\Theta^{*}}\left(x\right)$$

Inhere, each *f*_Θ*_(*x*) represents an SVR model optimized by Optuna.

Bagging is the averaging of multiple SVR models by running them in parallel within an AdaBoost framework.

Multiple AdaBoost SVR models can be trained by Bagging

7 $$F_{bagging}\left(x\right)=\frac{1}{B}\Sigma_{b=1}^{B}F_{b}\left(x\right)$$

where *B* is the number of different SVR models which used in Bagging and *F*_*b*_(*x*) represents the estimation of each submodel.

For each model, a weighted estimate is calculated as follows

8 $$ŷ=\frac{1}{B}\Sigma_{b=1}^{B}\Sigma_{m=1}^{M}\alpha_{m}^{\left(b\right)}f_{m}^{\left(b\right)}\left(x\right)$$

where α_*m*_^(*b*)^ is the weight of the weak learner *m* in model *b* in Bagging, *f*_*m*_^(*b*)^(*x*) is the prediction model *m* of model *b*. Through Bagging, the generalization performance of the AdaBoost SVR model increases while the variance decreases.^54,56,57^

#### 2.2.2 Gradient Boosting

The boosting algorithm was first proposed by Valiant and is based on the idea of combining multiple weak learners to create a strong model.^58^ After that, Friedman developed the Gradient Boosting algorithm to optimize nonparametric prediction models.^59^ This method is called Gradient Boosting Regression Tree (GBRT) and uses Decision Trees (DTs) as base learners. GBRT minimizes a loss function in the direction of the steepest descent at each step to improve the model incrementally.^47,48^ This model calculates the residual errors of previous predictions and creates new decision trees to minimize these errors in every iteration. As new nodes are created, the loss function of the model is adjusted to decrease further. In the case that the performance of the model is not sufficient, the process is repeated to determine the optimal split points and correct the errors.

The general structure of the model is as follows: (Liu and Zhang)

9 $$f\left(x\right)=\sum_{m=1}^{M}\hat{C_{m}}I\left(x\in R_{m}\right)$$

where *f*(*x*) represents the estimated function, $\hat{C_{m}}$ is the weighted coefficient for each region (*R_m_*), *I*(*x* ∈ *R_m_*) is the indicator function showing whether the *x* sample belongs to the *R_m_* region.

Estimating the mean prediction

10 $$\hat{C_{m}}=\frac{1}{N}\Sigma_{x_{i}\in R_{m\left(j,s\right)}}y_{i}$$

where $\hat{C_{m}}$ is the average predicted value, *N* is the number of samples, *y_i_* is the set of true values, and *R*_*m*(*j*,*s*)_ is the data set divided into regions.

Optimization of error minimization

11 $$\min_{j,s}\left[\min_{c_{1}}\Sigma_{x_{i}\in R_{1\left(j,s\right)}}{\left(y_{i}-c_{1}\right)}^{2}+\min_{c_{2}}\Sigma_{x_{i}\in R_{2\left(j,s\right)}}{\left(y_{i}-c_{2}\right)}^{2}\right]$$

where *c*_1_, *c*_2_ are the parameters determining the best regional estimates, *y_i_* are the true values, and *R*_1(*j*,*s*)_, *R*_2(*j*,*s*)_ are the regionalized subsets of the data set.

The model divides the data set into small decision trees and minimizes the error at each iteration using optimization. To improve the model performance, hyperparameters such as learning rate, number of estimators, tree depth, minimum samples split, minimum leaf size, and sample ratio were optimized by the RandomizedSearchCV method. This method randomly searches for different combinations of parameters and determines the configuration that gives the optimal results.

#### 2.2.3 Multilayer Perceptron (MLP)

Multilayer Perceptron (MLP) is a type of artificial neural network (ANN) commonly used in supervised learning problems such as classification and regression. MLP that consists of input, hidden, and output layers works by having each layer’s neurons acting upon the data of the previous layer with activation functions and transferring it to the next layer. The most commonly used activation functions are Rectified Linear Unit (ReLU), Sigmoid (logsig), Hyperbolic Tangent (tansig), Linear (purelin), and Sinusid. These functions provide the model to learn nonlinear relationships. In this study, ReLU activation function was preferred to learn nonlinear relationships in hidden layers more efficiently and because of its widespread use in regression-based neural network applications.^50,60^ In addition, ReLU has advantages over traditional saturating activation functions such as sigmoid. Sigmoid function may negatively affect learning performance at very high or very low input values.^61,62^

In case of the MLP model having a single output and single hidden layer, the equation is obtained as in the formula below

12 $$ỹ=\delta_{2}\left(\Sigma_{i=1}^{m}(w_{i}^{\left(2\right)}\delta_{1}\left(X\right)\right)+b^{\left(2\right)})X=\Sigma_{j=1}^{n}\left(x_{j}w_{\mathrm{xj}}^{\left(1\right)}\right)+b^{\left(1\right)}$$

where the symbol *ỹ* is the predicted vector of the MLP model, and this value is determined based on the input feature vectors, represented *x*_*j*_. Also, *w* ^(2)^ is the weight connecting the output layer to the hidden layer. *w* ^(1)^ is the weight connecting the input layer to the hidden layer. The output layer uses an activation function called δ_2_. *m* and *n* represent the number of samples (observations) and the number of features (variables) in the data set, respectively. The neurons in the hidden layer are activated with the δ_1_ function. *b* ^(1)^ and *b* ^(2)^ symbols represent the bias vectors used in the hidden layer and output layer.

Moreover, to obtain an optimum performance, some parameters such as solver functions, activation functions, and hidden layer dimensions must be adjusted. These parameters are called hyperparameters and require fine-tuning. The Optuna framework is used to tune these hyperparameters efficiently. Optuna is a simple and useful library for hyperparameter optimization that automatically optimizes hyperparameters such as learning rate, regularization coefficient (alpha), and hidden layer structure. Although activation function and solver can also be optimized using Optuna, in this study, they were manually fixed as ReLU and Adam, respectively, to ensure training stability and reduce computational load. Optuna automates the searching process with Bayesian optimization and tree-structured Parzen estimator (TPE) algorithms. Thereby, this can ensure high accuracy and low computational cost. MLP models are usually trained with back-propagation and gradient descent algorithms. Also, algorithms such as Bayesian regularization, Levenberg–Marquardt (LM), and scaled conjugate gradient are effectively used to optimize the weights and bias terms during training.

Consequently, the combination of MLP’s flexible learning capacity and Optuna’s efficient hyperparameter searching capability significantly increases model accuracy and overall efficiency.^49–51,60,63^

#### 2.2.4 Voting Regressor

The Voting Regressor method is an ensemble technique that merges the predictions of multiple base learners by means of linear combination. In this method, there are two basic averaging strategies which are employed. These are simple averaging and weighted averaging. Simple Averaging Ensemble (SAE), also known as unweighted or naïve averaging, is the most widely used averaging method especially in models such as artificial neural networks. SAE generates the final prediction result by taking the arithmetic average of predictions of all base learners. This method can be applied to both regression and classification problems with some limitations. For instance, weak learners or overconfident predictions can proportionately influence the overall outcome. The Weighted Averaging Ensemble (WAE) works by assigning a weight to each base learner’s prediction which is determined according to the model’s performance. WAE generally achieves better prediction accuracy, calibration and validity when compared to the SAE method.^64–66^

The formulations of the SAE and WAE Voting Regressor model can be expressed as follows

13 $$ŷ=\frac{1}{n}\Sigma_{i=1}^{n}{ŷ}_{İ}$$

Equation 13 gives the basic equation for the SAE Voting Regressor method.

Here, the symbol *ŷ* is the final (ensemble) prediction. *n* is the total number of base models (learners) and $\hat{y_{İ}}$ indicates the predicted value produced by *i*-th base model (e.g., MLP, SVR, GBR)

14 $$ŷ=\Sigma_{i=1}^{n}w_{i}{ŷ}_{İ}$$

Equation 14 presents the fundamental equation for the WAE Voting Regressor method.

In this formula, the symbol *ŷ* again refers to is the final (ensemble) prediction. *w*_*i*_ is the weight assigned to each base model, $\hat{y_{İ}}$ denotes the predicted value of the *i*-th model and *n* indicates the total number of models used.^67^

In our approach, the predictions of Optuna-optimized MLP, AdaBoost SVR, and Gradient Boosting models were combined using the Voting Regressor algorithm based on Weighted Averaging Ensemble (WAE). In this method, the weights of each base learners were optimized using the Optuna framework in order to maximize the model’s cross-validated *R* ^2^ score. Compared to SAE, the WAE provides better predictive performance and adaptability, although it requires additional computation due to the optimization process. Therefore, a WAE model was preferred to construct a high performance and generalizable ensemble regressor.

## 3 Results and Discussion

Herein, hyperparameters for each model were optimized by using a genetic algorithm. Each hyperparameter combination was evaluated by considering different subsets of model training and testing, and the parameter combination that gave the best result for each model was identified. In Table 2, optimal hyperparameter results were presented for each model that was obtained through the genetic algorithm.

**Table 2:** Optimal Hyperparameters for Each Model

model	optimal hyperparameters
Optuna AdaBoost SVR	base_estimator = SVR (C = 9.976, epsilon = 0.0442, kernel = “rbf”), n_estimators = 360, learning_rate = 0.054, loss = “square”
Bagging AdaBoost SVR	base_estimator = SVR (C = 1.1943, epsilon = 0.00056, kernel = “linear”), n_estimators = 338, learning_rate = 0.10417, loss = “square”
Gradient Boosting	n_estimators = 200, learning_rate = 0.05, max_depth = 3, min_samples_split = 10, min_samples_leaf = 2, subsample = 0.6
Optuna MLP	hidden_layer_1′: 135, ’hidden_layer_2′: 181, activation = “relu”, solver = “adam” α = 0.00488, learning_rate_init = 0.001089
Voting Regressor	weights = (1.9257; 0.3447; 0.3098); base models: (Gradient Boosting; MLP; AdaBoost(SVR))

Additionally, various statistical performance metrics, including determination coefficient (*R* ^2^), mean square error (MSE), and mean absolute percentage error (MAPE), were used to assess and compare the prediction performances of the machine learning models developed. The mathematical expressions and definitions of these metrics are given below:^68^

- Determination coefficient (*R* ^2^)­
15 $$R^{2}=1-\frac{\sum_{i=1}^{n}{\left(y_{i}-{ŷ}_{i}\right)}^{2}}{\sum_{i=1}^{n}{\left(y_{i}-\mathrm{y̅}\right)}^{2}}$$
  This metric represents how much of the total variance of the dependent variable that is explained by the independent variables. As the *R* ^2^ value approaches 1, the performance of the model improves.
- Mean square error (MSE)
16 $$\mathrm{MSE}=\frac{1}{n}\Sigma_{i=1}^{n}{\left(y_{i}-{ŷ}_{i}\right)}^{2}$$
  MSE gives the average of the squared differences between the model’s predicted value and the actual values. A lower MSE value indicates higher prediction accuracy.
- Mean absolute percentage error (MAPE)
17 $$\mathrm{MAPE}=\frac{100\%}{n}\Sigma_{i=1}^{n}\left|\frac{y_{i}-{ŷ}_{i}}{y_{i}}\right|$$

MAPE expresses the model’s predictive accuracy as a percentage, showing how close predictions are to the actual values on average.

In the above equations, *y*_*i*_ = actual observed value (oleic acid conversion).

$\hat{y_{i}}$ = predicted value (oleic acid conversion predicted by the model).

y̅ = the mean of actual values (average of actual oleic acid conversion).

*n* = number of data points.

To ensure model reproducibility, the random state = 42 parameter was fixed during both the train-test split and model training processes. The performance metrics were evaluated separately for the training and testing data sets to allow for a more detailed understanding of the models’ behavior. In addition, 5-fold cross-validation was applied to assess how well the models generalize to unseen data. For each fold, *R* ^2^, MSE, and MAPE values were computed, and the final cross-validation scores were obtained by averaging these results. Reporting training and testing results independently helps identify potential issues such as overfitting or underfitting, while also providing insights into the model’s learning capacity. Moreover, including cross-validation results enhances the reliability of the evaluation by demonstrating the stability of the model’s performance across different data subsets.^69^ All results are presented in Table 3.

**Table 3:** Results of Optuna-Optimized AdaBoost SVR, Bagging AdaBoost SVR, Gradient Boosting, Optuna-Optimized Dual-Layer MLP, and Voting Regressor Models

model	train *R* ^2^	test *R* ^2^	train MAPE	test MAPE	train MSE	test MSE	cross-validation *R* ^2^	cross-validation MAPE	cross-validation MSE
Optuna AdaBoost SVR	0.9860	0.9229	1.01%	3.40%	1.1735	10.5764	–0.2699	6.16%	42.2700
Bagging AdaBoost SVR	0.9000	0.9462	2.96%	3.16%	8.3647	7.3732	0.4943	4.91%	19.8453
Gradient Boosting	0.9883	0.9673	0.87%	2.21%	0.9825	4.4782	0.4257	5.74%	29.2312
Optuna MLP	0.9993	0.9846	0.16%	1.59%	0.0556	2.1163	0.5271	4.21%	17.2625
Voting Regressor	0.9933	0.9653	0.73%	2.27%	0.5567	4.7557	0.9513	1.76%	3.4221

Generally, test scores are prioritized in evaluating model performance, as they reflect the generalizability of the model to new and unseen data. However, evaluating both training and test scores is important to identify cases such as overfitting and underfitting.

As shown in Table 3, five different regression models were comparatively evaluated based on their training, test, and cross-validation performances using *R* ^2^, MAPE, and MSE metrics. The highest accuracy value (*R* ^2^ = 0.9846, MAPE = 1.59%) was achieved by the Optuna-optimized MLP model, but the cross-validation value (CV *R* ^2^ = 0.5271) fell behind the Voting Regressor model.

Among all models, the Voting Regressor model gave the most balanced and consistent results. This model obtained a high *R* ^2^ (0.9653) and a low MAPE (2.27%) on the test set while obtaining the highest cross-validation *R* ^2^ value (0.9513). These data demonstrated superior generalizability across all of the data layers. In addition, the lowest cross-validation MAPE (1.76%) and MSE (3.42) values supported the stability of the model.

On the other hand, although the Optuna AdaBoost SVR model gave acceptable results in the test set (*R* ^2^ = 0.9229), the negative cross-validation *R* ^2^ (−0.2699) shows that the model has high variance and low generalization ability. Although Gradient Boosting and Bagging AdaBoost SVR models showed reasonable and stable performances, they fell behind the Voting Regressor model based on overall success.

As a result, although the Optuna-optimized MLP was the most successful individual model in terms of test accuracy, the Voting Regressor model gave the best performance in both accuracy and stability when considering the overall success criteria. In this model, high generalizability and low error were achieved by combining three powerful models (MLP, Gradient Boosting, and AdaBoost SVR) previously optimized by Optuna. Therefore, it has been observed that the ensemble approach provides better results than the individual models.

The relation between the actual and predicted yield (%) for both training and test sets is presented in Figures 2–6. In all graphs, it can be seen that the majority of the data are clustered close to the ideal linear curve. This situation generally indicates that the models have high predictive performance. In particular, Optuna MLP and Voting Regressor models demonstrated closest distribution to the ideal linear line in both training and test sets which is also supported by their low error rates and high *R* ^2^ scores. The Gradient Boosting model also showed a very successful distribution in the training data; however, deviating from the curve in some extreme points of test set implies that the model may have limited generalizability despite its overall accuracy. On the other hand, in Bagging AdaBoost SVR and Optuna AdaBoost SVR models, it can be seen that the points in the test set are relatively far from the ideal line because of having higher error rates compared to other models.

**Figure 2:**
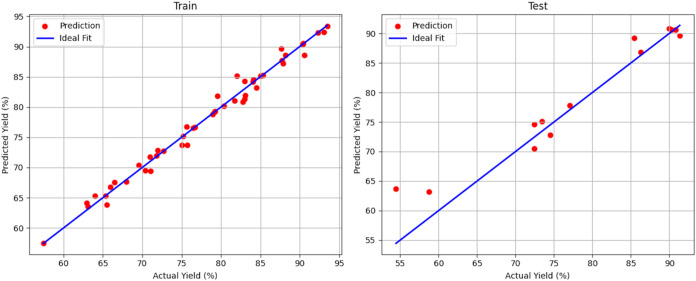
Correlation plots between actual and predicted yields for training and testing data using Optuna AdaBoost SVR.

**3:**
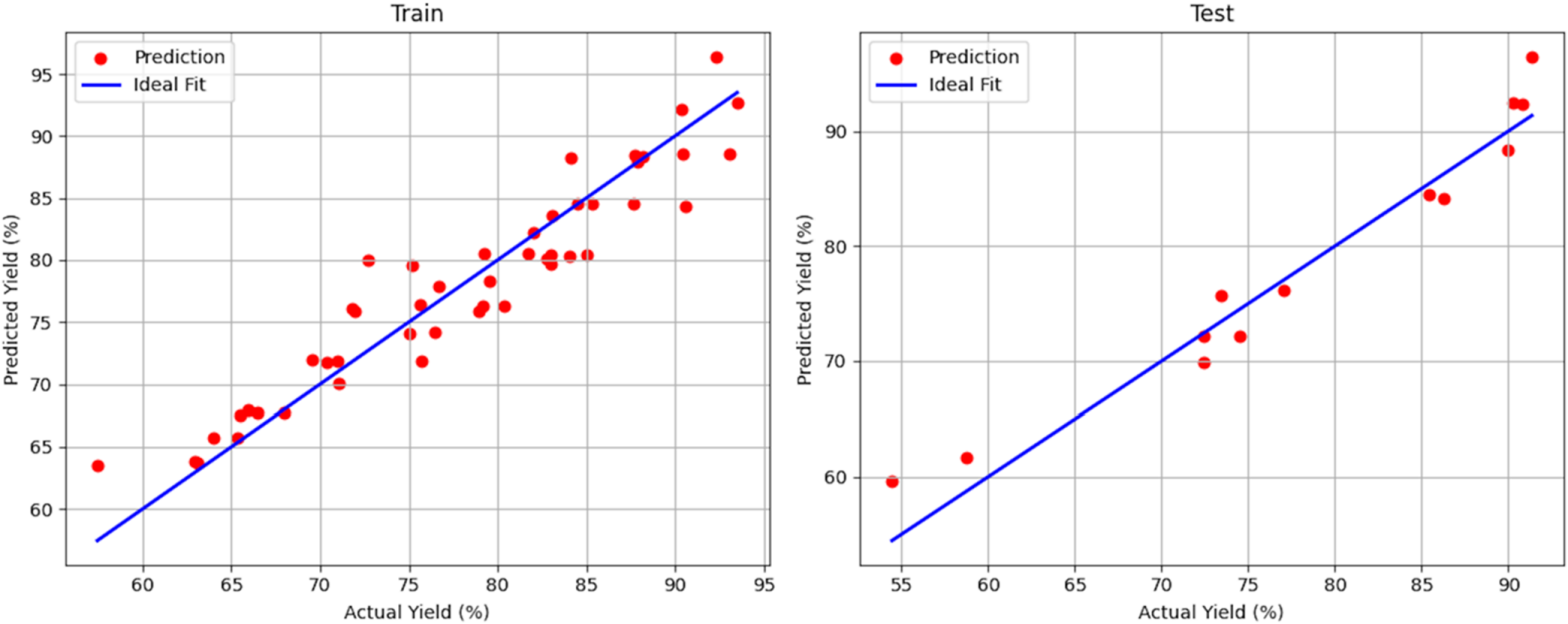
Correlation plots between actual and predicted yields for training and testing data using Bagging AdaBoost SVR.

**4:**
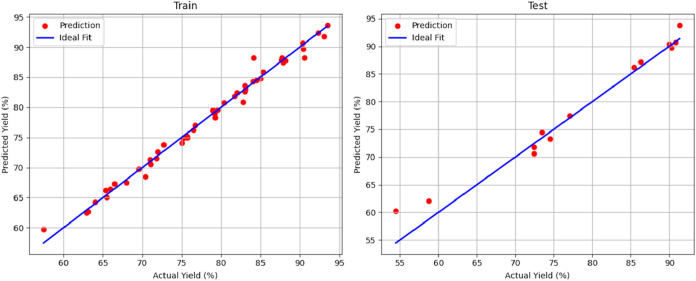
Correlation plots between actual and predicted yields for training and testing data using Gradient Boosting.

**5:**
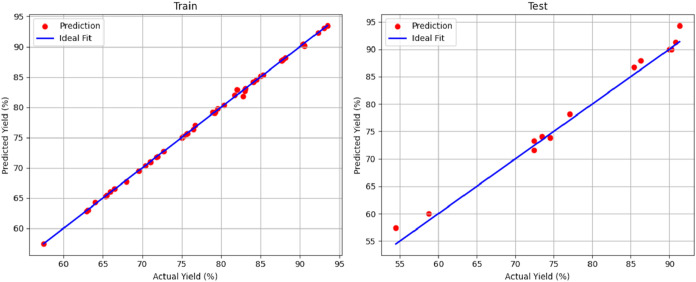
Correlation plots between actual and predicted yields for training and testing data using Optuna MLP.

**Figure 6:**
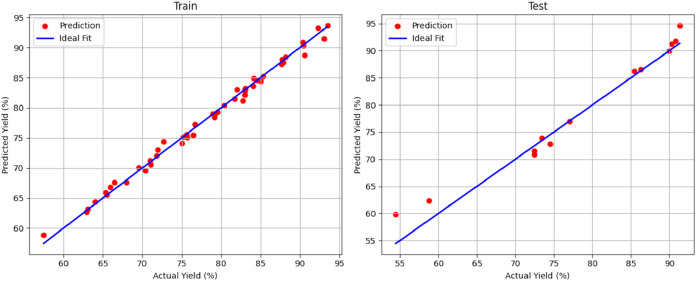
Correlation plots between actual and predicted yields for training and testing data using Voting Regressor.

As a result, graphical comparisons support the numerical performance metrics and demonstrate that especially Optuna MLP and Voting Regressor models yielded stable and accurate results.

Since the reaction temperature remained constant during the experiments, the model was not trained on temperature variations. Therefore, response surface plots that included temperature were excluded, and only the effects of the remaining parameters were evaluated. The parameters of catalyst concentration, reaction time, and the methanol/oleic acid molar ratio have significant effects on the oleic acid conversion in esterification reactions. Therefore, in Figures 7–9, the effects of these parameters on oleic acid conversion are shown with 3D surface plots obtained with the binary effects of the input variables. Figure 7 shows that increasing the catalyst amount has a positive effect on oleic acid conversion. However, after reaching 12–14 wt % catalyst amount, there was a slowdown in the rate of increase in conversion, showing a plateau tendency at the 18% level. The reason for this is the mass transfer rate or contact rate between the catalyst, methanol, and oleic acid reaching the equilibrium point. In other words, it is evaluated as the increasing amount of catalyst restricting the contact between the reactants in the reaction medium.^70–72^ Similarly, as the reaction time increased, the oleic acid conversion increased. Especially at low catalyst amounts, the effect of extending reaction time was more clearly observed. However, after 5 h of reaction time, the slope of increase began to decline. This situation indicates that economical catalyst usage and reaction time should be evaluated together in optimization studies.

**Figure 7:**
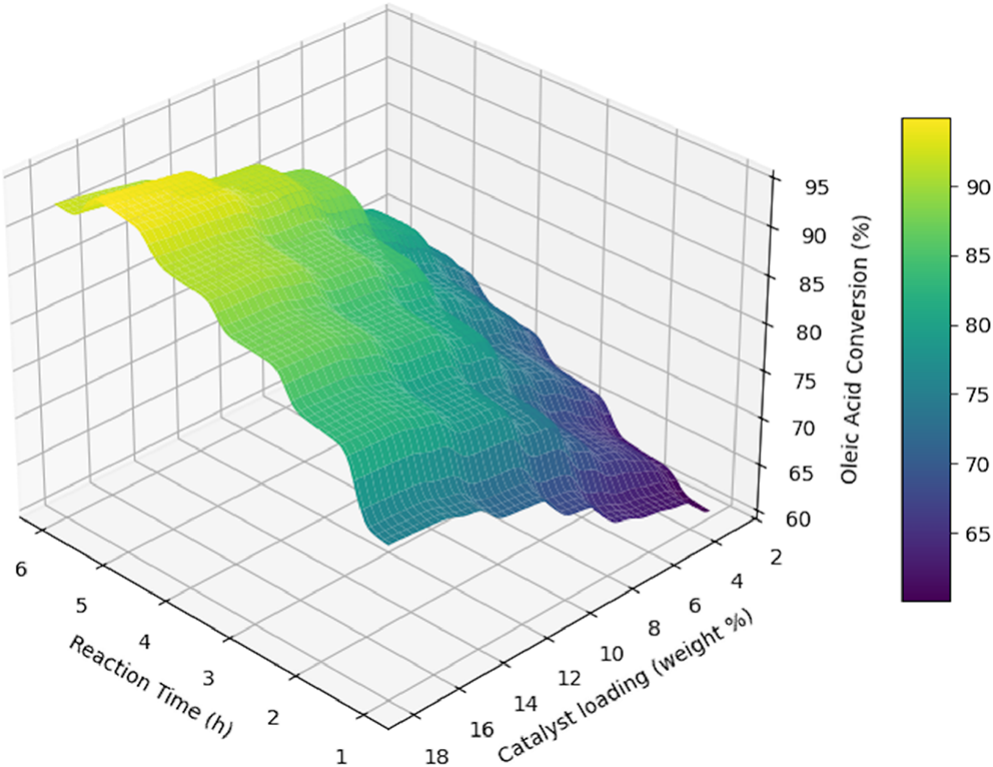
Effect of reaction time (h) and catalyst loading (wt %) on the oleic acid conversion predicted by Voting Regressor (Temperature: 67 °C and methanol/oleic acid molar ratio: 6). Optimized amount of oleic acid conversion (%) = 94.96 if reaction time = 5.29 h and catalyst amount (wt %) = 18.

**Figure 8:**
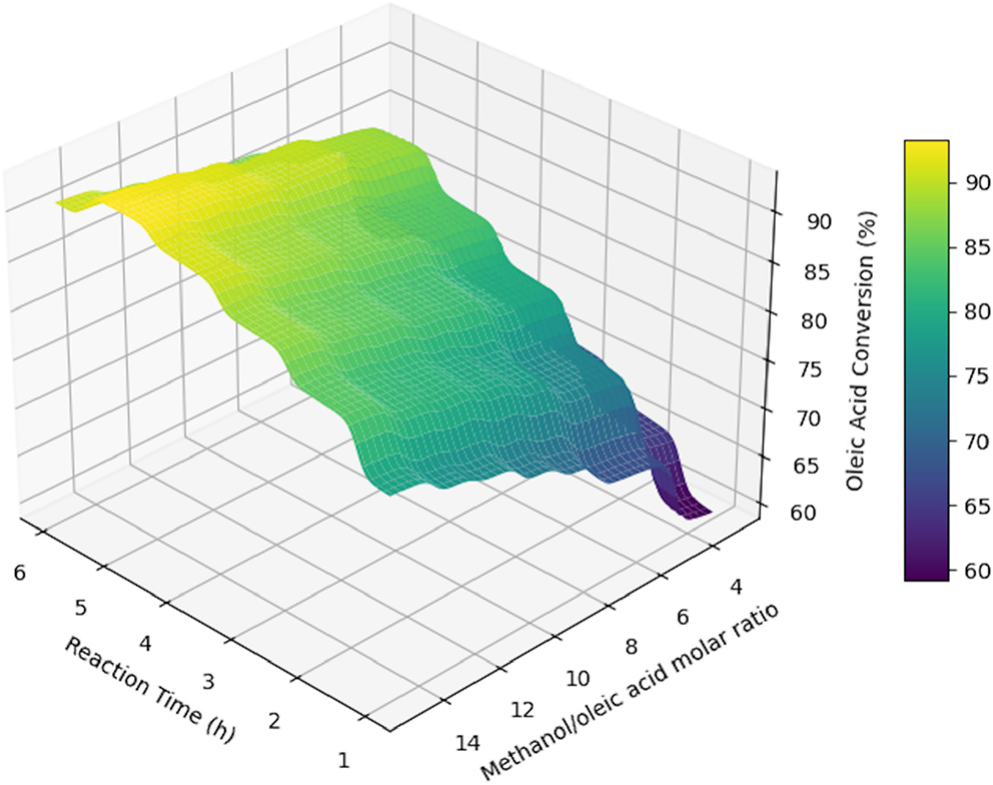
Effect of reaction time (h) and methanol/oleic acid molar ratio on the oleic acid conversion predicted by Voting Regressor (temperature: 67 °C and catalyst loading (wt %): 9). Optimized amount of oleic acid conversion (%) = 93.30 if reaction time = 5.24 h and methanol/oleic acid molar ratio = 15.

**Figure 9:**
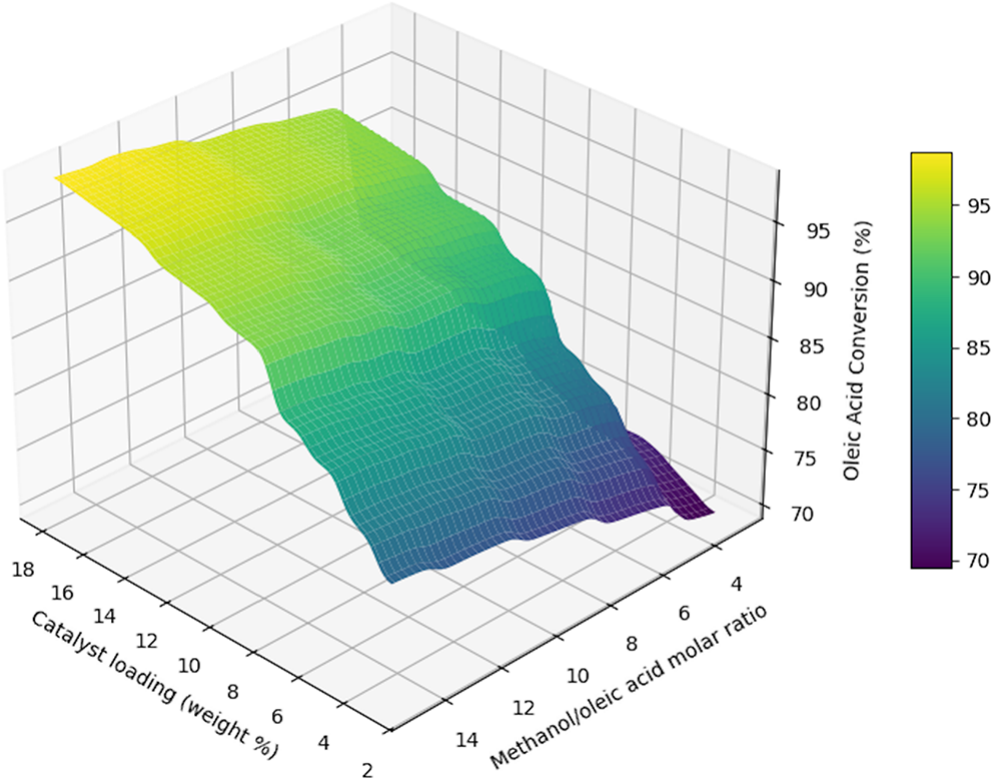
Effect of catalyst loading (wt %) and the methanol/oleic acid molar ratio on the oleic acid conversion predicted by Voting Regressor (temperature: 67 °C and reaction time: 5 h). Optimized amount of oleic acid conversion (%) = 98.79 if methanol/oleic acid molar ratio = 15 and catalyst amount (wt %) = 18.

In Figure 8, an increase in oleic acid conversion was observed as the methanol/oil molar ratio increased from 3:1 to 10:1. However, the rate of increase slowed down after reaching a 10:1 methanol/oleic acid molar ratio. Such behavior is commonly observed in reversible esterification reactions. Excessive methanol can have adverse effects on solubility or equilibrium.^73,74^ Moreover, the use of excessive alcohol is not preferred due to its requirement of higher energy for the recovery process and increment of the industrial production cost of methyl ester.^75^ Reaction time had an enhancing effect on the oleic acid conversion, and the trend slowed down after 5 h of reaction time. This suggests that excessive methanol makes a limited contribution to oleic acid conversion and that the optimum reaction time is around 5 h.

Figure 9 presents the influence of catalyst amount (wt %) and methanol/oleic acid molar ratio on oleic acid conversion. The increase in the amount of catalyst caused a significant increment in the conversion rate, especially between 2 and 10 wt % catalyst amount. However, an increase above 12 wt % catalyst amount decreased the growth rate, and a tendency to plateau was observed on the surface. This situation indicates the occurrence of saturation effect at high catalyst amount. Similarly, as the methanol/oleic acid molar ratio increased, the conversion rate also increased, but this increment was more limited than the catalyst amount increase. The effect of the methanol/oleic acid molar ratio tended to plateau after about a 12:1 molar ratio. Therefore, after a certain point, the conversion increment in both parameters became stagnant, which shows that chemical yield and optimum economic conditions should be evaluated together.

The individual effects of each reaction parameter on the oleic acid conversion are presented in Figures 10–12. In each 2-dimensional graph, one of the reaction parameters was changed at regular intervals while the other parameters remained constant. As shown in the graphs, the increase up to a certain point in all parameter amounts increased the oleic acid conversion. Approximately after 5 h of reaction time, a decline in oleic acid conversion was observed, while after a certain point, the increase in oleic acid conversion started to decrease with increasing catalyst amount and the methanol/oleic acid molar ratio. These findings are consistent with the results presented in Figures 7–9 as earlier demonstrated.

**Figure 10:**
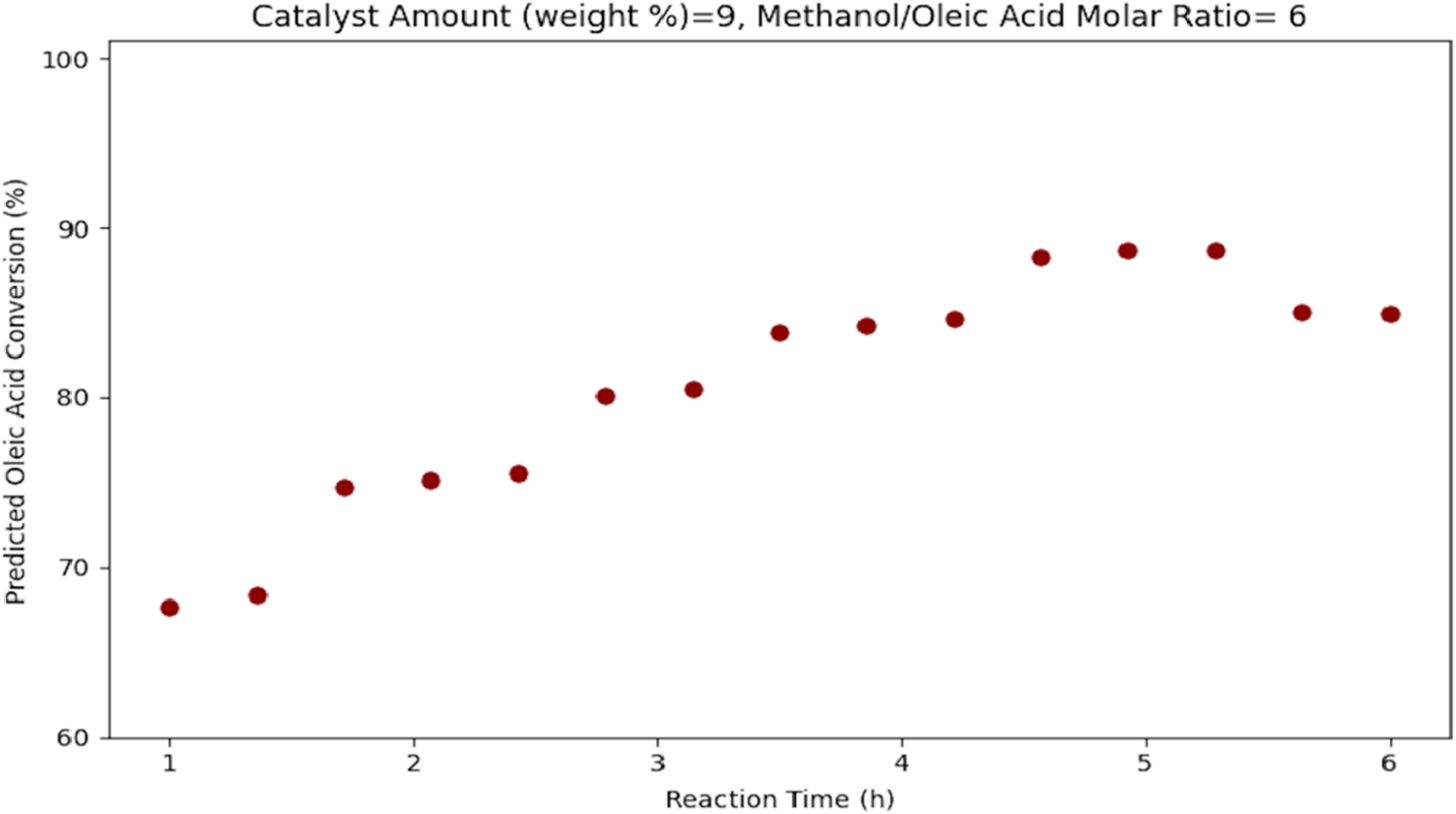
Individual effect of reaction time on oleic acid conversion estimated by the Voting regressor.

**11:**
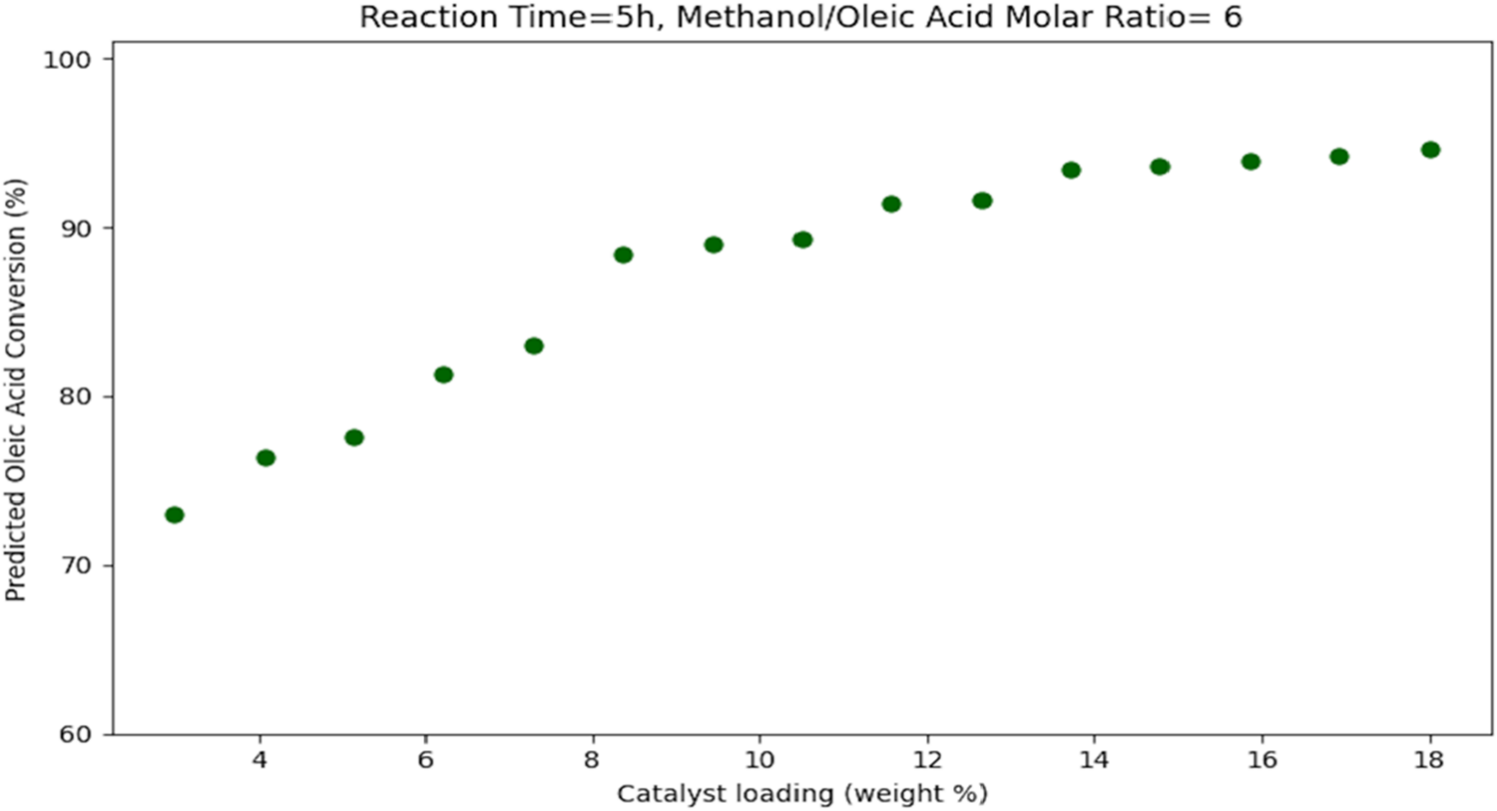
Individual effect of catalyst loading on oleic acid conversion estimated by the Voting regressor.

**Figure 12:**
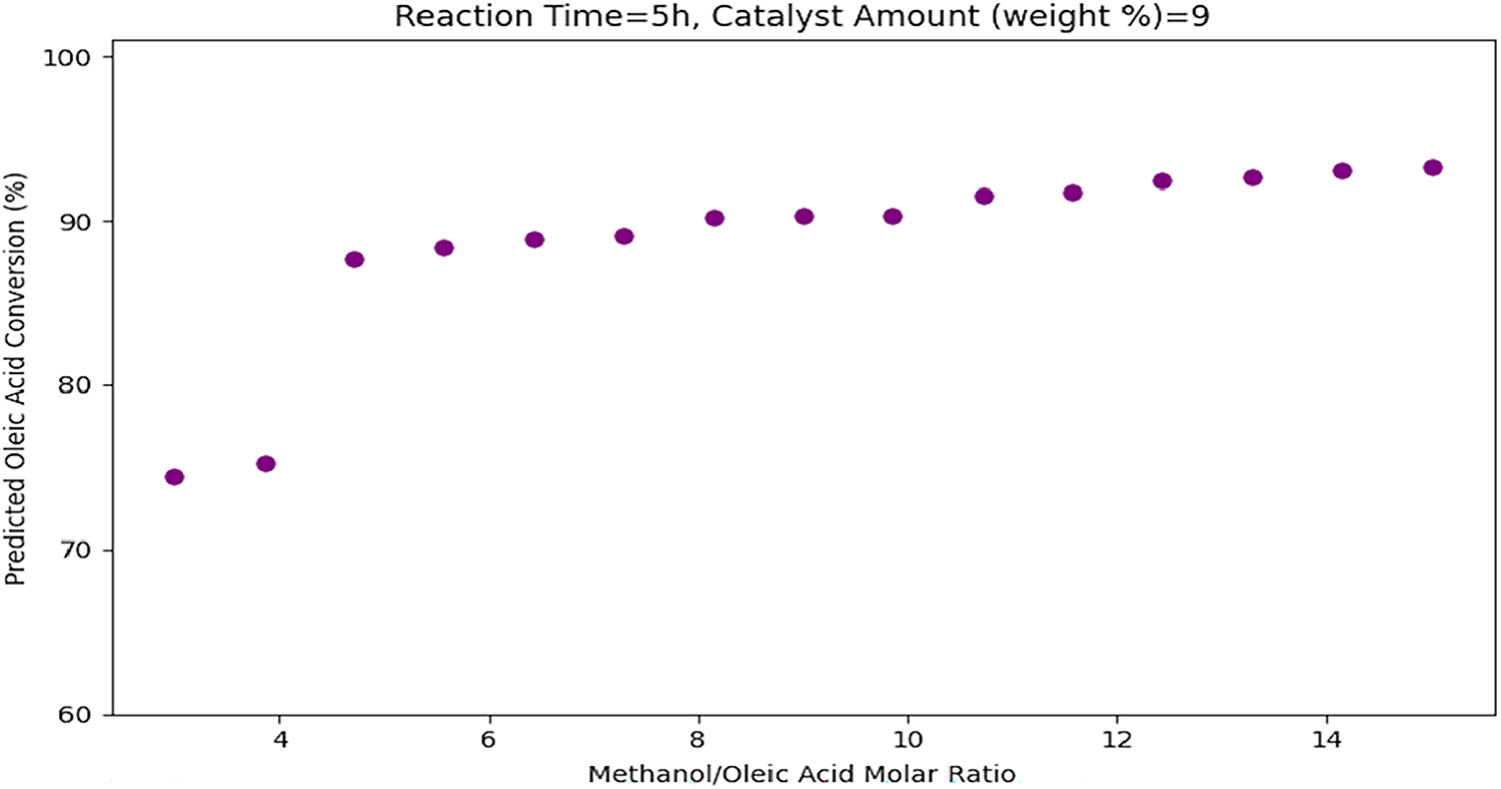
Individual effect of methanol/oleic acid molar ratio on oleic acid conversion estimated by the Voting regressor.

In order to maximize oleic acid conversion, the optimum value of each parameter was determined by the Voting Regressor model. Table 4 shows the results of the optimization process. Based on these findings, the maximum yield obtained for oleic acid conversion was 99.23%. This value was obtained under the conditions of 67 °C reaction temperature, 5.49 h reaction time, 18 (wt %) catalyst amount, and 15 methanol/oleic acid molar ratio.

**Table 4:** Optimum Parameter Values for Maximum Response at 67°C

*X*_2_ = reaction time (h)	*X*_3_ = catalyst loading (wt %)	*X*_4_ = methanol/oleic acid molar ratio	*Y* = oleic acid conversion
5.49	18	15:1	99.23

## 4 Conclusions

In this study, the biodiesel production process based on the esterification reaction of oleic acid was modeled and optimized with different machine learning algorithms. In the modeling process, MLP, AdaBoost SVR, Gradient Boosting, and Voting Regressor algorithms, which is a combination of the first three models, were applied. The performance of the models was measured by some criteria such as MSE, MAPE, and *R* ^2^. The Voting Regressor model was found to demonstrate high performance due to the high train and test *R* ^2^ (0.9933, 0.9653) and low MAPE (0.73%, 2.27%) and MSE (0.5567, 4.7557) values obtained on the training and test data. In addition, at a similar level of the 5-fold cross-validation results indicated that the model did not show an overfitting tendency and had high generalization ability. Especially the highest *R* ^2^ (0.9513) and lowest error rates (MAPE = 1.76%, MSE = 3.4221) obtained in the cross-validation phase made the Voting Regressor model more reliable compared to other models and were selected as the final model.

The MLP model optimized with Optuna attracted attention among the single models with the highest train and test *R* ^2^ (0.9993, 0.9846) and lowest MAPE (0.16%, 1.59%) and MSE (0.0556, 2.1163) for train and test values and the Gradient Boosting model with the low MSE (4.4782) in the test set. The Bagging AdaBoost SVR model showed significant performance, especially with a high *R* ^2^ (0.9462) obtained in the test data. These findings showed that the Voting Regressor, as an ensemble model, produced statistically more balanced and reliable results, while the other algorithms were also competitive in certain metrics.

In the multiple regression analysis performed with the Voting Regressor model, surface graphs were obtained, and individual variable effects were examined separately. According to the results obtained, the oleic acid conversion rate increased as the catalyst amount and methanol/oleic acid molar ratio increased; however, these increases reached saturation after a certain point and tended to plateau. After about 5 h of reaction time, a decrease in oleic acid conversion was observed. Furthermore, in the parameter analysis performed with the Voting Regressor model, the optimum conditions were determined as 5.49 h reaction time, 18% catalyst loading, and a 15:1 methanol/oleic acid molar ratio, with an oleic acid conversion of 99.23% being estimated under these conditions.

In conclusion, this study aims to obtain more stable and accurate predictions by using an ensemble (Voting Regressor) method that combines different machine learning algorithms, rather than being limited to a single modeling method. Input data were obtained experimentally, and due to the model created with these data, optimization decisions related to the process were supported with graphs. Especially in biodiesel production based on esterification reaction, the use of such detailed regression analysis and surface plots is quite limited in the literature. In this respect, the study provides a new and an original contribution to the field in both forecasting and decision-making based on data.

The findings of this study showed that ensemble-learning-assisted modeling can reduce experimental effort, time, and operational cost in esterification-based biodiesel processes. In addition, the proposed approach may be adapted to various feedstocks and process conditions in terms of industrial biodiesel production applications and may support process optimization in chemical engineering systems.

Therefore, future studies may focus on applying the proposed model to different esterification systems, catalyst types, and larger experimental data sets in order to evaluate its generalization capacity and industrial applicability.

## Supplementary Material



## Notes

Python scripts for model training, hyperparameter optimization, and performance evaluation of the Gradient Boosting, AdaBoost SVR, Voting Regressor, and Optuna-optimized MLP models; experimental data set used for machine learning modeling and prediction of biodiesel production via esterification (PDF)
