# Supplementary material for: Comparing Ensemble and Standalone Machine Learning Models for Optimizing Biodiesel Production via Esterification
Source: ACS Omega. 2026 Jun 26;11(27):40653–66. doi: 10.1021/acsomega.6c04115 (PMC13382738; doi:10.1021/acsomega.6c04115)
Supplement: Supplementary file 1 [file ao6c04115_si_001.pdf]

SUPPORTING INFORMATION

Comparing Ensemble and Standalone Machine Learning Models for Optimizing Biodiesel Production via Esterification

Melike İmge Şenoymak Tarakçı [1,*]
[1]Faculty of Engineering (B), Chemical Engineering Departmant, Kocaeli University, Umuttepe Campus, 41380, İzmit- Kocaeli/Turkey
*Corresponding Author, email: imge.senoymak@kocaeli.edu.tr

Script S1. Gradient Boosting Model

```
from sklearn.ensemble import GradientBoostingRegressor
from sklearn.model_selection import RandomizedSearchCV, train_test_split,
cross_val_score
from sklearn.preprocessing import StandardScaler
from sklearn.metrics import r2_score, mean_squared_error, make_scorer
import pandas as pd
import numpy as np

# Veri Yükleme
df = pd.read_csv("esterifikasyon_verileri.csv")

# Methanol/Oleic Acid oranını sayısala dönüştürme
df['Methanol/oleic acid molar ratio'] = df['Methanol/oleic acid molar
ratio'].apply(
    lambda x: float(x.split('/')[0]) / float(x.split('/')[1])
)

# Giriş ve çıkış değişkenlerini belirleme
X = df[['Temperature (˚C)', 'Reaction Time (h)', 'Catalyst loading (weight
%)', 'Methanol/oleic acid molar ratio']]
y = df['Oleic acid conversion(%)']

# Veriyi ölçeklendirme
scaler = StandardScaler()
X_scaled = scaler.fit_transform(X)

# Eğitim ve test setine ayırma
X_train, X_test, y_train, y_test = train_test_split(X_scaled, y,
test_size=0.2, random_state=42)

# Gradient Boosting Modeli
gbr_model = GradientBoostingRegressor(random_state=42)

# Hiperparametre Aralıkları
```

```
param_distributions = {
    'n_estimators': [50, 100, 200],
    'learning_rate': [0.01, 0.05, 0.1, 0.2],
    'max_depth': [3, 5, 7],
    'min_samples_split': [2, 5, 10],
    'min_samples_leaf': [1, 2, 4],
    'subsample': [0.6, 0.8, 1.0]
}

# Randomized Search
random_search = RandomizedSearchCV(
    estimator=gbr_model,
    param_distributions=param_distributions,
    n_iter=30,
    scoring='r2',
    cv=5,
    random_state=42,
    n_jobs=-1
)

# Modeli Eğitme
random_search.fit(X_train, y_train)

# En İyi Model
best_model = random_search.best_estimator_

# Performans Değerlendirme
y_train_pred = best_model.predict(X_train)
y_test_pred = best_model.predict(X_test)

train_r2 = r2_score(y_train, y_train_pred)
test_r2 = r2_score(y_test, y_test_pred)
train_mse = mean_squared_error(y_train, y_train_pred)
test_mse = mean_squared_error(y_test, y_test_pred)

# MAPE Hesaplama Fonksiyonu
```

```
def mean_absolute_percentage_error(y_true, y_pred):
    return np.mean(np.abs((y_true - y_pred) / y_true)) * 100

train_mape = mean_absolute_percentage_error(y_train, y_train_pred)
test_mape = mean_absolute_percentage_error(y_test, y_test_pred)

# Çapraz Doğrulama Skorları
cv_r2_scores = cross_val_score(best_model, X_scaled, y, cv=5, scoring='r2',
n_jobs=-1)
cv_mse_scores = -cross_val_score(best_model, X_scaled, y, cv=5,
scoring='neg_mean_squared_error', n_jobs=-1)
cv_mape_scores = cross_val_score(
    best_model, X_scaled, y, cv=5,
    scoring=make_scorer(mean_absolute_percentage_error,
greater_is_better=False),
    n_jobs=-1
)

# Sonuçları Yazdırma
print(f"\n✅ En İyi Parametreler: {random_search.best_params_}")
print(f"✅ Train R² Skoru: {train_r2:.4f}")
print(f"✅ Test R² Skoru: {test_r2:.4f}")
print(f"✅ Train MSE: {train_mse:.4f}")
print(f"✅ Test MSE: {test_mse:.4f}")
print(f"✅ Train MAPE: {train_mape:.2f}%")
print(f"✅ Test MAPE: {test_mape:.2f}%")
print(f"✅ Çapraz Doğrulama Ortalama R² Skoru:
{np.mean(cv_r2_scores):.4f}")
print(f"✅ Çapraz Doğrulama Ortalama MSE Skoru:
{np.mean(cv_mse_scores):.4f}")
print(f"✅ Çapraz Doğrulama Ortalama MAPE Skoru: {-
np.mean(cv_mape_scores):.2f}%")
```

Script S2. AdaBoost SVR Bagging Model

```
from sklearn.ensemble import BaggingRegressor, AdaBoostRegressor
from sklearn.svm import SVR
from sklearn.model_selection import cross_val_score, train_test_split
from sklearn.preprocessing import StandardScaler
from sklearn.metrics import r2_score, mean_squared_error
import numpy as np
import pandas as pd

# 📌 **Veri Yükleme**
df = pd.read_csv("esterifikasyon_verileri.csv")

# 📌 **Methanol/Oleic Acid Oranını Sayısala Dönüştürme**
df['Methanol/oleic acid molar ratio'] = df['Methanol/oleic acid molar
ratio'].apply(
    lambda x: float(x.split('/')[0]) / float(x.split('/')[1])
)

# 📌 **Giriş ve Çıkış Değişkenlerini Belirleme**
X = df[['Temperature (˚C)', 'Reaction Time (h)', 'Catalyst loading (weight
%)', 'Methanol/oleic acid molar ratio']]
y = df['Oleic acid conversion(%)']

# 📌 **Veriyi Ölçeklendirme**
scaler = StandardScaler()
X_scaled = scaler.fit_transform(X)

# 📌 **Eğitim ve Test Setlerine Ayırma**
X_train, X_test, y_train, y_test = train_test_split(X_scaled, y,
test_size=0.2, random_state=42)

# 📌 **En İyi Optuna AdaBoost SVR Modeli Parametreleri**
best_adaboost_svr = AdaBoostRegressor(
    estimator=SVR(kernel='linear', C=1.1943, epsilon=0.00056),
```

```
    n_estimators=338,
    learning_rate=0.10417,
    loss='square',
    random_state=42
)

# 📌 **Bagging ile AdaBoost SVR Modeli**
bagging_adaboost = BaggingRegressor(
    estimator=best_adaboost_svr,  # ✅ base_estimator yerine estimator
kullanılıyor
    n_estimators=20,  # 🔥 Daha fazla estimator ile genelleme artar
    max_samples=0.8,   # 🔥 %80 veri ile eğitim
    max_features=1.0,  # 🔥 Tüm özellikler kullanılacak
    bootstrap=True,
    n_jobs=-1,
    random_state=42
)

# 📌 **Modeli Eğitme**
bagging_adaboost.fit(X_train, y_train)

# 📌 **Tahmin Yapma**
y_train_pred = bagging_adaboost.predict(X_train)
y_test_pred = bagging_adaboost.predict(X_test)

# 📌 **Performans Değerlendirme**
train_r2 = r2_score(y_train, y_train_pred)
test_r2 = r2_score(y_test, y_test_pred)

train_mse = mean_squared_error(y_train, y_train_pred)
test_mse = mean_squared_error(y_test, y_test_pred)

train_mape = np.mean(np.abs((y_train - y_train_pred) / y_train)) * 100
test_mape = np.mean(np.abs((y_test - y_test_pred) / y_test)) * 100
```

```
# 📌 **Çapraz Doğrulama**
cv_r2_scores = cross_val_score(bagging_adaboost, X_scaled, y, cv=5,
scoring='r2', n_jobs=-1)
cv_mse_scores = -cross_val_score(bagging_adaboost, X_scaled, y, cv=5,
scoring='neg_mean_squared_error', n_jobs=-1)
cv_mape_scores = cross_val_score(bagging_adaboost, X_scaled, y, cv=5,
scoring=lambda model, X, y: np.mean(np.abs((y - model.predict(X)) / y)) *
100, n_jobs=-1)

# 📌 **Sonuçları Yazdırma**
print(f"✅ Bagging AdaBoost SVR Train R² Skoru: {train_r2:.4f}")
print(f"✅ Bagging AdaBoost SVR Test R² Skoru: {test_r2:.4f}")
print(f"✅ Bagging AdaBoost SVR Train MSE: {train_mse:.4f}")
print(f"✅ Bagging AdaBoost SVR Test MSE: {test_mse:.4f}")
print(f"✅ Bagging AdaBoost SVR Train MAPE: {train_mape:.2f}%")
print(f"✅ Bagging AdaBoost SVR Test MAPE: {test_mape:.2f}%")

print(f"\n✅ Bagging AdaBoost SVR Çapraz Doğrulama R² Skorları:
{cv_r2_scores}")
print(f"✅ Bagging AdaBoost SVR Ortalama Çapraz Doğrulama R² Skoru:
{np.mean(cv_r2_scores):.4f}")

print(f"✅ Bagging AdaBoost SVR Çapraz Doğrulama MSE Skorları:
{cv_mse_scores}")
print(f"✅ Bagging AdaBoost SVR Ortalama Çapraz Doğrulama MSE Skoru:
{np.mean(cv_mse_scores):.4f}")

print(f"✅ Bagging AdaBoost SVR Çapraz Doğrulama MAPE Skorları:
{cv_mape_scores}")
print(f"✅ Bagging AdaBoost SVR Ortalama Çapraz Doğrulama MAPE Skoru:
{np.mean(cv_mape_scores):.2f}%")
```

Script S3. AdaBoost SVR Optuna Model

```
import optuna
from sklearn.svm import SVR
from sklearn.ensemble import AdaBoostRegressor
from sklearn.model_selection import train_test_split, cross_val_score
from sklearn.preprocessing import StandardScaler
from sklearn.metrics import r2_score, mean_squared_error,
mean_absolute_percentage_error
import pandas as pd
import numpy as np

# 🔷 Veri Yükleme
df = pd.read_csv("esterifikasyon_verileri.csv")

# 🔷 Methanol/oleic acid oranını sayısal değere çevirme
df['Methanol/oleic acid molar ratio'] = df['Methanol/oleic acid molar
ratio'].apply(
    lambda x: float(x.split('/')[0]) / float(x.split('/')[1])
)

# 🔷 Giriş ve çıkış değişkenlerini ayırma
X = df[['Temperature (˚C)', 'Reaction Time (h)', 'Catalyst loading (weight
%)', 'Methanol/oleic acid molar ratio']]
y = df['Oleic acid conversion(%)']

# 🔷 Ölçeklendirme
scaler = StandardScaler()
X_scaled = scaler.fit_transform(X)

# 🔷 Eğitim ve test veri seti ayırma
X_train, X_test, y_train, y_test = train_test_split(X_scaled, y,
test_size=0.2, random_state=42)

# 🔷 Optuna optimizasyon fonksiyonu
```

```
def objective(trial):
    C = trial.suggest_float("C", 0.1, 10.0)
    epsilon = trial.suggest_float("epsilon", 1e-4, 0.1)
    kernel = trial.suggest_categorical("kernel", ["linear", "rbf"])
    loss = trial.suggest_categorical("loss", ["linear", "square",
"exponential"])
    n_estimators = trial.suggest_int("n_estimators", 300, 400)
    learning_rate = trial.suggest_float("learning_rate", 0.05, 0.15)

    svr = SVR(C=C, epsilon=epsilon, kernel=kernel)
    model = AdaBoostRegressor(
        estimator=svr,
        n_estimators=n_estimators,
        learning_rate=learning_rate,
        loss=loss,
        random_state=42
    )

    scores = cross_val_score(model, X_train, y_train, cv=5, scoring="r2",
n_jobs=-1)
    return scores.mean()

# 🔷 Optuna çalıştırma (tekrarlanabilirlik için seed eklendi)
from optuna.samplers import TPESampler

study = optuna.create_study(
    direction="maximize",
    sampler=TPESampler(seed=42)  # Rastgeleliği sabitler
)
study.optimize(objective, n_trials=50)

# 🔷 En iyi parametreleri alma
best_params = study.best_params
print("✅ En İyi Parametreler:", best_params)

# 🔷 En iyi modelin kurulması
```

```
best_svr = SVR(C=best_params["C"], epsilon=best_params["epsilon"],
kernel=best_params["kernel"])
best_model = AdaBoostRegressor(
    estimator=best_svr,
    n_estimators=best_params["n_estimators"],
    learning_rate=best_params["learning_rate"],
    loss=best_params["loss"],
    random_state=42
)

# 🔷 Modeli eğitme
best_model.fit(X_train, y_train)

# 🔷 Tahminler
y_train_pred = best_model.predict(X_train)
y_test_pred = best_model.predict(X_test)

# 🔷 Performans metrikleri
train_r2 = r2_score(y_train, y_train_pred)
test_r2 = r2_score(y_test, y_test_pred)
train_mse = mean_squared_error(y_train, y_train_pred)
test_mse = mean_squared_error(y_test, y_test_pred)
train_mape = mean_absolute_percentage_error(y_train, y_train_pred) * 100
test_mape = mean_absolute_percentage_error(y_test, y_test_pred) * 100

# 🔷 5 katlı çapraz doğrulama metrikleri
cv_r2_scores = cross_val_score(best_model, X_scaled, y, cv=5, scoring='r2')
cv_mse_scores = -cross_val_score(best_model, X_scaled, y, cv=5,
scoring='neg_mean_squared_error')
cv_mape_scores = cross_val_score(
    best_model, X_scaled, y, cv=5,
    scoring=lambda est, X, y: mean_absolute_percentage_error(y,
est.predict(X)),
    n_jobs=-1
)
```

```
# 🔷 Sonuçları yazdırma
print(f"\n✅ Train R²: {train_r2:.4f}")
print(f"✅ Test R²: {test_r2:.4f}")
print(f"✅ Train MSE: {train_mse:.4f}")
print(f"✅ Test MSE: {test_mse:.4f}")
print(f"✅ Train MAPE: {train_mape:.2f}%")
print(f"✅ Test MAPE: {test_mape:.2f}%")

print("\n📌 Çapraz Doğrulama R² Skorları:", cv_r2_scores)
print("📌 Ortalama CV R²:", np.mean(cv_r2_scores))
print("📌 Çapraz Doğrulama MSE Skorları:", cv_mse_scores)
print("📌 Ortalama CV MSE:", np.mean(cv_mse_scores))
print("📌 Çapraz Doğrulama MAPE Skorları:", cv_mape_scores)
print("📌 Ortalama CV MAPE:", np.mean(cv_mape_scores) * 100, "%")
```

Script S4. Optuna MLP Model

```
import optuna
from sklearn.neural_network import MLPRegressor
from sklearn.model_selection import train_test_split, cross_val_score
from sklearn.preprocessing import StandardScaler
from sklearn.metrics import r2_score, mean_squared_error
import numpy as np
import pandas as pd
from optuna.samplers import TPESampler

# 📌 **Veri Yükleme**
df = pd.read_csv("esterifikasyon_verileri.csv")

# **Methanol/Oleic Acid oranını sayısala dönüştürme**
df['Methanol/oleic acid molar ratio'] = df['Methanol/oleic acid molar
ratio'].apply(
    lambda x: float(x.split('/')[0]) / float(x.split('/')[1])
)

# **Giriş ve çıkış değişkenlerini belirleme**
X = df[['Temperature (˚C)', 'Reaction Time (h)', 'Catalyst loading (weight
%)', 'Methanol/oleic acid molar ratio']]
y = df['Oleic acid conversion(%)']

# **Veriyi ölçeklendirme**
scaler = StandardScaler()
X_scaled = scaler.fit_transform(X)

# **Eğitim ve test setine ayırma**
X_train, X_test, y_train, y_test = train_test_split(X_scaled, y,
test_size=0.2, random_state=42)

# **Optuna ile Optimizasyon Fonksiyonu**
def objective(trial):
    hidden_layer_1 = trial.suggest_int('hidden_layer_1', 32, 256)
```

```
    hidden_layer_2 = trial.suggest_int('hidden_layer_2', 32, 256)
    alpha = trial.suggest_loguniform('alpha', 1e-4, 1e-2)
    learning_rate_init = trial.suggest_loguniform('learning_rate_init', 1e-
4, 1e-2)

    # **MLP Modeli**
    mlp_model = MLPRegressor(
        hidden_layer_sizes=(hidden_layer_1, hidden_layer_2),
        activation='relu',
        solver='adam',
        alpha=alpha,
        learning_rate_init=learning_rate_init,
        max_iter=5000,
        random_state=42
    )

    # **Çapraz doğrulama ile değerlendirme**
    scores = cross_val_score(mlp_model, X_scaled, y, cv=5, scoring='r2',
n_jobs=-1)
    return np.mean(scores)

# **Optuna Çalıştırma**
study = optuna.create_study(direction='maximize')
study.optimize(objective, n_trials=30)

# **En İyi Parametreleri Alma**
best_params = study.best_params
print("\n✅ En İyi Parametreler:", best_params)

# **En İyi Modeli Eğitme**
best_mlp = MLPRegressor(
    hidden_layer_sizes=(best_params['hidden_layer_1'],
best_params['hidden_layer_2']),
    activation='relu',
    solver='adam',
    alpha=best_params['alpha'],
```

```
    learning_rate_init=best_params['learning_rate_init'],
    max_iter=5000,
    random_state=42
)

best_mlp.fit(X_train, y_train)

# **Model Performansı**
y_train_pred = best_mlp.predict(X_train)
y_test_pred = best_mlp.predict(X_test)

train_r2 = r2_score(y_train, y_train_pred)
test_r2 = r2_score(y_test, y_test_pred)
train_mse = mean_squared_error(y_train, y_train_pred)
test_mse = mean_squared_error(y_test, y_test_pred)

def mean_absolute_percentage_error(y_true, y_pred):
    return np.mean(np.abs((y_true - y_pred) / y_true)) * 100

train_mape = mean_absolute_percentage_error(y_train, y_train_pred)
test_mape = mean_absolute_percentage_error(y_test, y_test_pred)

# **Çapraz Doğrulama Sonuçları**
cv_scores = cross_val_score(best_mlp, X_scaled, y, cv=5, scoring='r2',
n_jobs=-1)
cv_mse_scores = -cross_val_score(best_mlp, X_scaled, y, cv=5,
scoring='neg_mean_squared_error', n_jobs=-1)
cv_mape_scores = cross_val_score(
    best_mlp, X_scaled, y, cv=5,
    scoring=lambda model, X, y: mean_absolute_percentage_error(y,
model.predict(X)),
    n_jobs=-1
)

# **Sonuçları Yazdırma**
print(f"\n✅ Train R² Skoru: {train_r2:.4f}")
```

```
print(f"✅ Test R² Skoru: {test_r2:.4f}")
print(f"✅ Train MSE: {train_mse:.4f}")
print(f"✅ Test MSE: {test_mse:.4f}")
print(f"✅ Train MAPE: {train_mape:.2f}%")
print(f"✅ Test MAPE: {test_mape:.2f}%")
print("\n✅ Çapraz Doğrulama R² Skorları:", cv_scores)
print(f"✅ Ortalama Çapraz Doğrulama R² Skoru: {cv_scores.mean():.4f}")
print("✅ Çapraz Doğrulama MSE Skorları:", cv_mse_scores)
print(f"✅ Ortalama Çapraz Doğrulama MSE Skoru:
{cv_mse_scores.mean():.4f}")
print("✅ Çapraz Doğrulama MAPE Skorları:", cv_mape_scores)
print(f"✅ Ortalama Çapraz Doğrulama MAPE Skoru:
{np.mean(cv_mape_scores):.2f}%")
```

Script S5. Voting Regressor (opt_mlp_svr_gra) Model

```
import numpy as np
import optuna
from sklearn.ensemble import VotingRegressor, GradientBoostingRegressor,
AdaBoostRegressor
from sklearn.neural_network import MLPRegressor
from sklearn.svm import SVR
from sklearn.model_selection import train_test_split, cross_val_score
from sklearn.preprocessing import StandardScaler
from sklearn.metrics import r2_score, mean_squared_error,
mean_absolute_percentage_error
import pandas as pd
from optuna.samplers import TPESampler

# 📌 Veri Setini Yükleme
df = pd.read_csv("esterifikasyon_verileri.csv")

# 📌 Methanol/Oleic Acid oranını sayısala dönüştürme
df['Methanol/oleic acid molar ratio'] = df['Methanol/oleic acid molar
ratio'].apply(
    lambda x: float(x.split('/')[0]) / float(x.split('/')[1])
)

# 📌 Giriş ve Çıkış Değişkenlerini Ayırma
X = df[['Temperature (˚C)', 'Reaction Time (h)', 'Catalyst loading (weight
%)', 'Methanol/oleic acid molar ratio']]
y = df['Oleic acid conversion(%)']

# 📌 Veriyi Ölçeklendirme
scaler = StandardScaler()
X_scaled = scaler.fit_transform(X)

# 📌 Eğitim ve Test Setine Ayırma
```

```
X_train, X_test, y_train, y_test = train_test_split(X_scaled, y,
test_size=0.2, random_state=42)

# 📌 En iyi AdaBoost SVR Modelini Tanımlama
best_adaboost_svr = AdaBoostRegressor(
    estimator=SVR(kernel="linear", C=1.1943, epsilon=0.00056),
    n_estimators=338,
    learning_rate=0.1041,
    loss="square",
    random_state=42
)

# 📌 En iyi Gradient Boosting Modelini Tanımlama
best_gb = GradientBoostingRegressor(
    n_estimators=168,
    max_depth=4,
    min_samples_split=12,
    min_samples_leaf=1,
    learning_rate=0.1194,
    subsample=0.5177,
    random_state=42
)

# 📌 En iyi MLP Modelini Tanımlama
best_mlp = MLPRegressor(
    hidden_layer_sizes=(128, 128),
    activation="relu",
    solver="adam",
    alpha=0.01,
    max_iter=5000,
    random_state=42
)

# 📌 Voting Regressor Modeli
voting_regressor = VotingRegressor(
    estimators=[
```

```
        ('AdaBoost_SVR', best_adaboost_svr),
        ('Gradient_Boosting', best_gb),
        ('MLP', best_mlp)
    ],
    weights=[1, 1, 1]  # Optuna ile optimize edilecek
)

# 📌 Optuna ile Voting Ağırlıklarını Optimizasyon Fonksiyonu
def voting_fitness(trial):
    weight_adaboost = trial.suggest_float('weight_adaboost', 0.1, 3.0)
    weight_gb = trial.suggest_float('weight_gb', 0.1, 3.0)
    weight_mlp = trial.suggest_float('weight_mlp', 0.1, 3.0)

    # 📌 Voting Regressor Modelini Güncelleme
    voting_regressor.set_params(weights=[weight_adaboost, weight_gb,
weight_mlp])

    # 📌 Çapraz Doğrulama ile Değerlendirme
    scores = cross_val_score(voting_regressor, X_train, y_train, cv=5,
scoring="r2", n_jobs=-1)
    return scores.mean()  # 📌 Ortalama R² Skorunu Maksimize Et

# 📌 Optuna Optimizasyonu Başlatma
study = optuna.create_study(
    direction="maximize",
    sampler=TPESampler(seed=42)
)
study.optimize(voting_fitness, n_trials=30)

# 📌 En İyi Ağırlıkları Çıktılama
best_weights = study.best_params
print(f"✅ En İyi Voting Ağırlıkları: {best_weights}")

# 📌 En İyi Voting Modelini Yeniden Eğitme
```

```
voting_regressor.set_params(weights=[best_weights['weight_adaboost'],
best_weights['weight_gb'], best_weights['weight_mlp']])
voting_regressor.fit(X_train, y_train)

# 📌 Model Performansını Test Setinde Değerlendirme
y_train_pred = voting_regressor.predict(X_train)
y_test_pred = voting_regressor.predict(X_test)

train_r2 = r2_score(y_train, y_train_pred)
test_r2 = r2_score(y_test, y_test_pred)

train_mse = mean_squared_error(y_train, y_train_pred)
test_mse = mean_squared_error(y_test, y_test_pred)

train_mape = mean_absolute_percentage_error(y_train, y_train_pred) * 100
test_mape = mean_absolute_percentage_error(y_test, y_test_pred) * 100

print(f"✅ Train R² Skoru: {train_r2:.4f}")
print(f"✅ Test R² Skoru: {test_r2:.4f}")
print(f"✅ Train MSE: {train_mse:.4f}")
print(f"✅ Test MSE: {test_mse:.4f}")
print(f"✅ Train MAPE: {train_mape:.2f}%")
print(f"✅ Test MAPE: {test_mape:.2f}%")

# 📌 Çapraz Doğrulama Performansını Hesaplama
cv_r2_scores = cross_val_score(voting_regressor, X_train, y_train, cv=5,
scoring="r2", n_jobs=-1)
cv_mse_scores = -cross_val_score(voting_regressor, X_train, y_train, cv=5,
scoring="neg_mean_squared_error", n_jobs=-1)
cv_mape_scores = cross_val_score(voting_regressor, X_train, y_train, cv=5,
scoring=lambda model, X, y: mean_absolute_percentage_error(y,
model.predict(X)) * 100, n_jobs=-1)

print(f"✅ Çapraz Doğrulama R² Skorları: {cv_r2_scores}")
print(f"✅ Ortalama Çapraz Doğrulama R² Skoru: {cv_r2_scores.mean():.4f}")
```

```
print(f"✅ Çapraz Doğrulama MSE Skorları: {cv_mse_scores}")
print(f"✅ Ortalama Çapraz Doğrulama MSE Skoru:
{cv_mse_scores.mean():.4f}")

print(f"✅ Çapraz Doğrulama MAPE Skorları: {cv_mape_scores}")
print(f"✅ Ortalama Çapraz Doğrulama MAPE Skoru:
{cv_mape_scores.mean():.2f}%")
```

Table 1

RUN	Temperature (C̆)	Reaction Time (h)	Catalyst loading (weight %)	Methanol/oleic acid molar ratio	Oleic acid conversion(%)
1	67	1	3	6/1	54.45
2	67	2	3	6/1	62.9
3	67	3	3	6/1	65.96
4	67	4	3	6/1	69.52
5	67	5	3	6/1	71.94
6	67	1	4.5	6/1	58.74
7	67	2	4.5	6/1	65.31
8	67	3	4.5	6/1	71.03
9	67	4	4.5	6/1	75
10	67	5	4.5	6/1	76.69
11	67	1	6	6/1	63.1
12	67	2	6	6/1	66.45
13	67	3	6	6/1	74.49
14	67	4	6	6/1	77.05
15	67	5	6	6/1	82.78
16	67	1	7.5	6/1	64
17	67	2	7.5	6/1	72.41
18	67	3	7.5	6/1	76.5
19	67	4	7.5	6/1	79.52
20	67	5	7.5	6/1	82.01
21	67	1	9	6/1	67.94
22	67	2	9	6/1	75.7
23	67	3	9	6/1	80.35
24	67	4	9	6/1	85.04
25	67	5	9	6/1	90.62
26	67	6	9	6/1	84.13
27	67	1	12	6/1	70.95
28	67	2	12	6/1	78.98
29	67	3	12	6/1	84.05
30	67	4	12	6/1	87.67
31	67	5	12	6/1	93.07
32	67	1	15	6/1	73.41
33	67	2	15	6/1	83.03
34	67	3	15	6/1	86.3
35	67	4	15	6/1	90
36	67	5	15	6/1	93.5
37	67	1	18	6/1	75.15
38	67	2	18	6/1	83.05
39	67	3	18	6/1	87.88

40	67	4	18	6/1	90.37
41	67	5	18	6/1	91.35
42	67	1	9	3/1	57.45
43	67	2	9	3/1	65.47
44	67	3	9	3/1	70.38
45	67	4	9	3/1	71.8
46	67	5	9	3/1	72.74
47	67	1	9	9/1	72.42
48	67	2	9	9/1	79.16
49	67	3	9	9/1	83
50	67	4	9	9/1	85.45
51	67	5	9	9/1	90.42
52	67	1	9	12/1	75.61
53	67	2	9	12/1	81.73
54	67	3	9	12/1	85.31
55	67	4	9	12/1	88.17
56	67	5	9	12/1	90.83
57	67	1	9	15/1	79.23
58	67	2	9	15/1	84.48
59	67	3	9	15/1	87.73
60	67	4	9	15/1	90.24
61	67	5	9	15/1	92.32
